# Supplementary material for: Transcriptomic analysis reveals similarities in genetic activation of detoxification mechanisms resulting from imidacloprid and chlorothalonil exposure
Source: PLoS One. 2018 Oct 25;13(10):e0205881. doi: 10.1371/journal.pone.0205881 (PMC6201883; doi:10.1371/journal.pone.0205881)
Supplement: S1 Fig — (PDF) [file pone.0205881.s001.pdf]

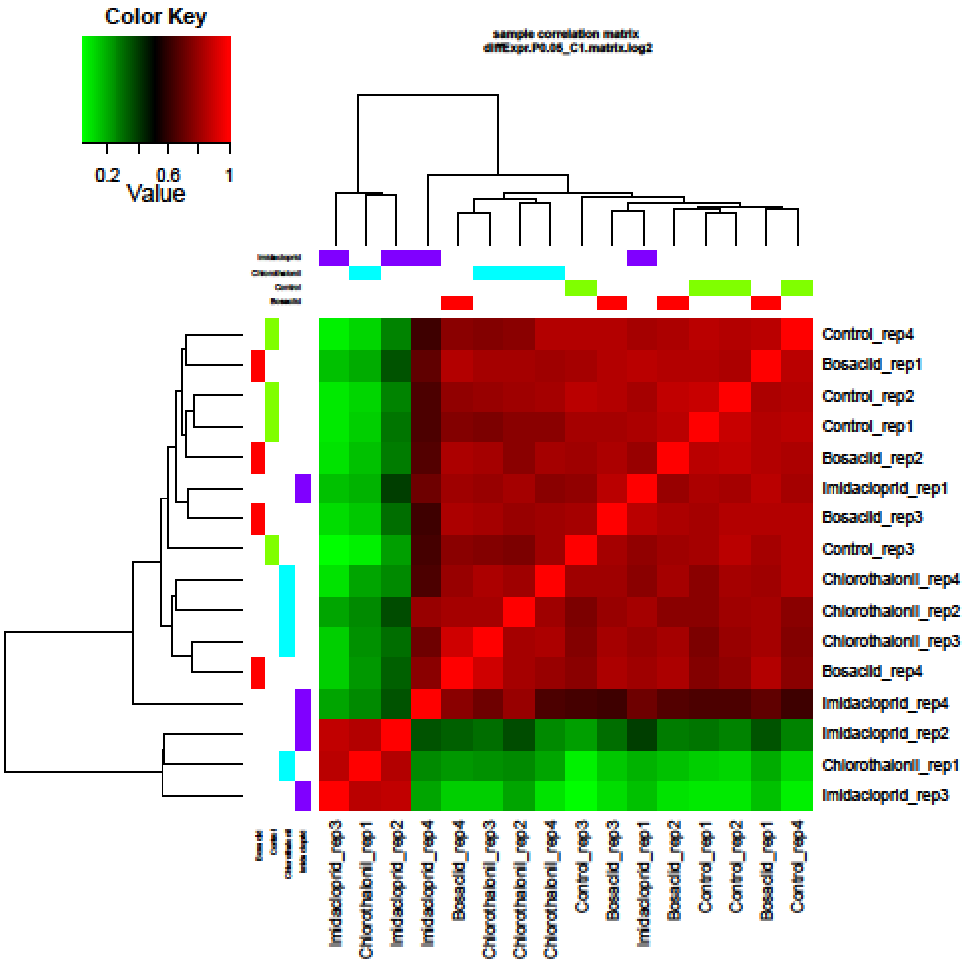

S1 Fig. Correlation matrix of transcript expression of beetles exposed to imidacloprid, boscalid, chlorothalonil and control.
