# Supplementary material for: Transcriptomic analysis reveals similarities in genetic activation of detoxification mechanisms resulting from imidacloprid and chlorothalonil exposure
Source: PLoS One. 2018 Oct 25;13(10):e0205881. doi: 10.1371/journal.pone.0205881 (PMC6201883; doi:10.1371/journal.pone.0205881)
Supplement: S1 Table — (PDF) [file pone.0205881.s002.pdf]

S1 Table. BLASTx and Blast2GO classification of Trinity transcripts of interest.

Blast2Go transcript designation	Trinity transcript ID	Sequences Producing Significant Alignments	Scientific Taxonomy	Sim	Hsp/ HIT	Hsp/ QUERY	NCBI Accession
**Acetylcholine receptor subunit alpha-like**	**DN52191_c2_g3**	acetylcholine receptor subunit alpha-like	Anoplophora glabripennis	50.80%	29.70%	45.60%	XP_018577209.1
**Carboxylesterase 5A**	**DN56141_c0_g1**	acetylcholinesterase	Aethina tumida	80.40%	74.50%	78.90%	XP_019869377.1
		carboxylesterase 5A	Anoplophora glabripennis	77.90%	77.30%	80.00%	XP_018566534.1
		fatty acyl-CoA hydrolase precursor, medium chain	Dendroctonus ponderosae	79.60%	73.20%	77.40%	XP_019754933.1
		acetylcholinesterase	Nicrophorus vespilloides	77.20%	75.60%	78.40%	XP_017772388.1
		pyrethroid hydrolase Ces2e	Tribolium castaneum	75.80%	74.80%	79.60%	XP_968892.1
**Cuticle 7-like**	**DN42933_c0_g1**	cuticle protein 19-like	Aethina tumida	71.40%	55.40%	35.10%	XP_019875899.1
		cuticle protein 7-like	Dendroctonus ponderosae	54.10%	88.70%	61.60%	XP_019763393.1
		cuticle protein 21-like	Dendroctonus ponderosae	55.40%	85.70%	60.20%	XP_019756770.1
		cuticle protein 7-like	Anoplophora glabripennis	84.40%	44.10%	22.90%	XP_018561021.1
		cuticle protein 19	Tribolium castaneum	76.70%	43.70%	26.20%	XP_968523.2
**Cuticle 7-like**	**DN45742_c0_g1**	cuticle protein 7-like	Anoplophora glabripennis	75.60%	83.20%	32.20%	XP_018561020.1
		cuticle protein 7-like	Aethina tumida	78.30%	72.30%	31.10%	XP_019875913.1
		cuticle protein 8-like	Aethina tumida	94.70%	47.80%	20.30%	XP_019875912.1
		cuticle protein 7-like	Dendroctonus ponderosae	93.20%	53.60%	20.00%	XP_019773288.1
		cuticle protein 8-like	Aethina tumida	95.90%	44.00%	20.00%	XP_019875898.1
**Cuticle-like**	**DN48928_c1_g1**	cuticle protein 16.5-like	Anoplophora glabripennis	80.70%	40.40%	9.90%	XP_018562781.1
		uncharacterized protein LOC108904860	Anoplophora glabripennis	78.90%	47.10%	9.90%	XP_018563075.1
		cuticle protein 16.5-like	Anoplophora glabripennis	77.20%	42.20%	9.90%	XP_018562756.1
		larval cuticle protein F1-like	Dendroctonus ponderosae	72.40%	33.50%	10.10%	XP_019764622.1
		cuticle protein 16.5-like	Anoplophora glabripennis	80.00%	40.70%	9.60%	XP_018562769.1
**Cytochrome P450 4C1-like isoform X1**	**DN45995_c0_g1**	cytochrome P450 4C1-like isoform X1	Anoplophora glabripennis	73.20%	90.00%	86.80%	XP_018572346.1
		PREDICTED: cytochrome P450 4C1-like isoform X2	Anoplophora glabripennis	71.80%	86.60%	77.60%	XP_018572356.1

Blast2Go transcript designation	Trinity transcript ID	Sequences Producing Significant Alignments	Scientific Taxonomy	Sim	Hsp/ HIT	Hsp/ QUERY	NCBI Accession
**Cytochrome P450 4C1-like isoform X1**	**DN45995_c0_g1**	cytochrome P450 4c3-like	Tribolium castaneum	61.60%	89.80%	88.80%	XP_015833627.1
		cytochrome P450 4C1	Tribolium castaneum	61.60%	89.50%	88.80%	XP_971612.1
		probable cytochrome P450 4d14	Tribolium castaneum	60.50%	90.30%	86.80%	XP_972577.1
**Cytochrome P450 4d2-like**	**DN46083_c0_g3**	cytochrome P450 monooxigenase CYP4H10	Tribolium castaneum	63.70%	94.90%	86.60%	NP_001107836.1
		cytochrome P450 4d2-like	Dendroctonus ponderosae	61.20%	95.80%	89.20%	XP_019760717.1
		cytochrome P450 4d2-like	Aethina tumida	61.00%	96.20%	88.30%	XP_019875047.1
		cytochrome P450 4d2-like	Anoplophora glabripennis	61.30%	94.40%	87.50%	XP_018565810.1
		cytochrome P450 4d2-like	Anoplophora glabripennis	61.30%	94.40%	87.50%	XP_018565811.1
**Cytochrome P450 6k1 isoform X1**	**DN61141_c1_g1**	cytochrome P450 6k1 isoform X1	Tribolium castaneum	66.50%	95.70%	24.70%	XP_970485.1
		cytochrome P450 6k1 isoform X1	Tribolium castaneum	66.50%	95.70%	24.70%	XP_008194912.1
		cytochrome P450 6k1 isoform X2	Tribolium castaneum	66.50%	95.70%	24.70%	XP_015835337.1
		cytochrome P450 6k1	Tribolium castaneum	61.90%	95.60%	24.60%	XP_970561.1
		cytochrome P450 6k1	Tribolium castaneum	61.20%	95.40%	24.60%	XP_970633.1
**Cytochrome P450 6k1-like**	**DN43906_c0_g1**	cytochrome P450 6k1-like	Anoplophora glabripennis	70.80%	100.00%	79.50%	XP_018561044.1
		cytochrome P450 6k1	Tribolium castaneum	71.00%	95.60%	76.40%	XP_970633.1
		cytochrome P450 6k1-like	Anoplophora glabripennis	68.80%	98.80%	79.10%	XP_018561043.1
		cytochrome P450 6k1	Tribolium castaneum	69.30%	100.00%	79.90%	XP_970561.1
		LOW QUALITY PROTEIN: cytochrome P450 6k1-like	Aethina tumida	70.00%	99.60%	79.10%	XP_019865070.1
**Endocuticle structural glyco bd-5-like**	**DN23859_c0_g1**	endocuticle structural glycoprotein SgAbd-5-like	Nicrophorus vespilloides	58.20%	60.70%	40.80%	XP_017785732.1
		larval cuticle protein 8	Tribolium castaneum	53.30%	68.20%	40.40%	XP_015836169.1
		endocuticle structural glycoprotein ABD-5-like	Anoplophora glabripennis	57.10%	66.40%	34.50%	XP_018576797.1
		larval cuticle protein LCP-17-like	Anoplophora glabripennis	53.20%	57.00%	34.50%	XP_018575118.1
		flexible cuticle protein 12-like	Anoplophora glabripennis	63.10%	47.80%	29.10%	XP_018568269.1
**Endocuticle structural glyco bd-8-like**	**DN53725_c1_g1**	endocuticle structural glycoprotein SgAbd-8-like	Anoplophora glabripennis	83.80%	104.50%	26.70%	XP_018578131.1

Blast2Go transcript designation	Trinity transcript ID	Sequences Producing Significant Alignments	Scientific Taxonomy	Sim	Hsp/ HIT	Hsp/ QUERY	NCBI Accession
**Endocuticle structural glyco bd-8-like**	**DN53725_c1_g1**	endocuticle structural glycoprotein SgAbd-8	Tribolium castaneum	76.50%	99.00%	28.30%	XP_973697.1
		uncharacterized protein LOC109542774	Dendroctonus ponderosae	76.90%	85.30%	26.90%	XP_019767705.1
		endocuticle structural glycoprotein SgAbd-8-like	Nicrophorus vespilloides	87.30%	75.00%	21.70%	XP_017785766.1
		endocuticle structural glycoprotein SgAbd-8-like	Aethina tumida	75.60%	86.30%	25.40%	XP_019867109.1
**Heat shock 68a**	**DN62524_c2_g1**	heat shock protein 68a	Tribolium castaneum	98.50%	50.70%	75.50%	NP_001164199.1
		heat shock protein 68b	Tribolium castaneum	98.50%	50.70%	75.50%	NP_001164200.1
		heat shock protein 70 A1	Tribolium castaneum	98.50%	50.60%	75.50%	XP_974442.1
		LOW QUALITY PROTEIN: heat shock protein 68-like	Aethina tumida	98.50%	50.90%	75.50%	XP_019874651.1
		major heat shock 70 kDa protein Ab	Anoplophora glabripennis	97.90%	62.40%	75.50%	XP_018574361.1
**heat shock 68-like**	**DN54580_c0_g1**	heat shock protein 68-like	Anoplophora glabripennis	95.40%	100.60%	65.40%	XP_018564820.1
		heat shock protein TC005094	Tribolium castaneum	93.80%	100.30%	65.40%	NP_001164098.1
		heat shock protein 68-like	Dendroctonus ponderosae	91.80%	100.50%	65.40%	XP_019771592.1
		major heat shock 70 kDa protein Ba-like	Agrilus planipennis	90.70%	100.00%	65.90%	XP_018322391.1
		LOW QUALITY PROTEIN: heat shock protein 68-like	Aethina tumida	90.00%	100.00%	66.50%	XP_019874651.1
**Heat shock 70 A1**	**DN41892_c0_g1**	heat shock protein 70 A1	Tribolium castaneum	97.60%	13.00%	99.20%	XP_974442.1
		heat shock protein 68a	Tribolium castaneum	97.60%	13.00%	99.20%	NP_001164199.1
		heat shock protein 68b	Tribolium castaneum	97.60%	13.00%	99.20%	NP_001164200.1
		heat shock protein 70 A1-like	Dendroctonus ponderosae	96.40%	12.80%	99.20%	XP_019760659.1
		heat shock protein 68-like	Dendroctonus ponderosae	97.60%	13.10%	99.20%	XP_019760660.1
**Heat shock 70 A1-like isoform X1**	**DN62524_c2_g2**	heat shock protein 70 A1-like isoform X1	Dendroctonus ponderosae	85.10%	35.10%	76.70%	XP_019760664.1
		heat shock protein 70 A1-like isoform X2	Dendroctonus ponderosae	85.10%	35.10%	76.70%	XP_019760665.1
		heat shock protein 70 A1-like	Agrilus planipennis	83.90%	25.80%	76.70%	XP_018336683.1
		LOW QUALITY PROTEIN: heat shock protein 68-like	Aethina tumida	86.90%	26.20%	76.70%	XP_019874651.1
		heat shock protein 68a	Tribolium castaneum	81.70%	26.20%	77.20%	NP_001164199.1

Blast2Go transcript designation	Trinity transcript ID	Sequences Producing Significant Alignments	Scientific Taxonomy	Sim	Hsp/ HIT	Hsp/ QUERY	NCBI Accession
**Heat shock 70 A1-like isoform X1**	**DN62524_c2_g4**	heat shock protein 70 A1-like isoform X1	Dendroctonus ponderosae	98.60%	14.40%	100.00%	XP_019760664.1
		heat shock protein 70 A1-like isoform X2	Dendroctonus ponderosae	98.60%	14.40%	100.00%	XP_019760665.1
		major heat shock 70 kDa protein Ab	Anoplophora glabripennis	100.00%	13.20%	100.00%	XP_018574361.1
		LOW QUALITY PROTEIN: heat shock protein 68-like	Aethina tumida	100.00%	10.70%	100.00%	XP_019874651.1
		heat shock protein 70 A1	Tribolium castaneum	100.00%	10.70%	100.00%	XP_974442.1
**Heat shock 70 kDa cognate 4-like**	**DN45929_c0_g1**	heat shock cognate 71 kDa protein isoform X1	Tribolium castaneum	86.00%	100.20%	86.00%	XP_015833401.1
		heat shock 70 kDa protein cognate 4-like	Dendroctonus ponderosae	86.10%	100.20%	85.60%	XP_019762664.1
		heat shock 70 kDa protein isoform X2	Tribolium castaneum	82.50%	105.10%	86.00%	XP_015833402.1
		heat shock cognate 71 kDa protein-like	Anoplophora glabripennis	89.70%	100.00%	77.70%	XP_018568222.1
		LOW QUALITY PROTEIN: heat shock 70 kDa protein cognate 4-like	Aethina tumida	83.80%	94.00%	81.50%	XP_019870154.1
**Multidrug resistance-associated 1 isoform X3**	**DN52951_c2_g1**	multidrug resistance-associated protein 1 isoform X3	Anoplophora glabripennis	74.70%	4.70%	30.00%	XP_018568572.1
		multidrug resistance-associated protein 1 isoform X4	Tribolium castaneum	74.70%	4.90%	30.00%	XP_008197317.1
		multidrug resistance-associated protein 1 isoform X9	Anoplophora glabripennis	76.00%	4.90%	30.00%	XP_018568578.1
		multidrug resistance-associated protein 1 isoform X2	Anoplophora glabripennis	76.00%	4.70%	30.00%	XP_018568571.1
		multidrug resistance-associated protein 1 isoform X3	Dendroctonus ponderosae	73.60%	4.80%	28.80%	XP_019754523.1
**Multidrug resistance-associated 4-like**	**DN48293_c3_g1**	probable multidrug resistance-associated protein lethal(2)03659	Anoplophora glabripennis	88.90%	47.10%	69.90%	XP_018574036.1
		multidrug resistance-associated protein 4-like	Aethina tumida	87.50%	43.10%	69.90%	XP_019877937.1
		multidrug resistance-associated protein 4-like	Anoplophora glabripennis	86.30%	43.10%	69.90%	XP_018561045.1
		multidrug resistance-associated protein 4-like	Anoplophora glabripennis	86.30%	43.10%	69.90%	XP_018561046.1
		multidrug resistance-associated protein 4-like	Anoplophora glabripennis	87.50%	43.00%	69.90%	XP_018575091.1

Blast2Go transcript designation	Trinity transcript ID	Sequences Producing Significant Alignments	Scientific Taxonomy	Sim	Hsp/ HIT	Hsp/ QUERY	NCBI Accession
**NADH-quinone oxidoreductase subunit B 2**	**DN33393_c0_g1**	uncharacterized protein LOC108907609	Anoplophora glabripennis	85.10%	92.90%	72.20%	XP_018566868.1
		NADH-quinone oxidoreductase subunit B 2-like	Nicrophorus vespilloides	91.40%	82.10%	60.40%	XP_017777433.1
		uncharacterized protein LOC109544473	Dendroctonus ponderosae	77.90%	99.60%	78.50%	XP_019770227.1
		NADH-quinone oxidoreductase subunit B 2	Tribolium castaneum	92.80%	82.20%	57.60%	XP_967271.1
		NADH-quinone oxidoreductase subunit B 2	Tribolium castaneum	92.80%	82.20%	57.60%	XP_008201665.1
**Probable cytochrome P450**	**DN45930_c0_g1**	probable cytochrome P450 49a1	Dendroctonus ponderosae	73.20%	94.10%	0.849	XP_019770320.1
		probable cytochrome P450 49a1, partial	Dendroctonus ponderosae	74.70%	101.20%	0.814	XP_019756292.1
		probable cytochrome P450 49a1, partial	Dendroctonus ponderosae	74.70%	101.40%	0.814	XP_019771175.1
		probable cytochrome P450 301a1, mitochondrial	Tribolium castaneum	73.10%	87.50%	0.793	XP_971083.1
		cytochrome P450 CYP12A2-like	Nicrophorus vespilloides	69.70%	91.70%	0.828	XP_017783894.1
**Probable cytochrome P450 305a1**	**DN51839_c1_g1**	uncharacterized protein LOC108913304	Anoplophora glabripennis	75.10%	46.40%	60.30%	XP_018574350.1
		probable cytochrome P450 305a1	Aethina tumida	73.70%	92.70%	59.00%	XP_019871644.1
		probable cytochrome P450 305a1	Tribolium castaneum	71.20%	96.30%	61.40%	XP_970235.1
		probable cytochrome P450 305a1	Nicrophorus vespilloides	69.50%	96.70%	61.60%	XP_017774949.1
		probable cytochrome P450 305a1	Agrilus planipennis	68.10%	91.10%	58.30%	XP_018325316.1
**Probable cytochrome P450**	**DN45930_c0_g1**	probable cytochrome P450 49a1	Dendroctonus ponderosae	73.20%	94.10%	84.90%	XP_019770320.1
		probable cytochrome P450 49a1, partial	Dendroctonus ponderosae	74.70%	101.20%	81.40%	XP_019756292.1
		probable cytochrome P450 49a1, partial	Dendroctonus ponderosae	74.70%	101.40%	81.40%	XP_019771175.1
		probable cytochrome P450 301a1, mitochondrial	Tribolium castaneum	73.10%	87.50%	79.30%	XP_971083.1
		cytochrome P450 CYP12A2-like	Nicrophorus vespilloides	69.70%	91.70%	82.80%	XP_017783894.1
**Probable cytochrome P450 49a1**	**DN47979_c8_g1**	probable cytochrome P450 49a1	Anoplophora glabripennis	89.30%	100.00%	89.20%	XP_018570997.1
		probable cytochrome P450 49a1	Anoplophora glabripennis	89.30%	100.00%	89.20%	XP_018570998.1
		probable cytochrome P450 49a1	Aethina tumida	88.50%	98.20%	86.00%	XP_019868560.1
		probable cytochrome P450 49a1	Dendroctonus ponderosae	87.70%	91.30%	85.50%	XP_019771989.1

Blast2Go transcript designation	Trinity transcript ID	Sequences Producing Significant Alignments	Scientific Taxonomy	Sim	Hsp/ HIT	Hsp/ QUERY	NCBI Accession
**Probable cytochrome P450 49a1**	**DN47979_c8_g1**	probable cytochrome P450 49a1	Nicrophorus vespilloides	87.10%	101.10%	87.90%	XP_017787115.1
**Probable cytochrome P450 4d14**	**DN46083_c0_g2**	probable cytochrome P450 4d14	Aethina tumida	64.90%	18.70%	9.30%	XP_019879051.1
		probable cytochrome P450 4d14	Aethina tumida	64.90%	18.70%	9.30%	XP_019879346.1
		cytochrome P450 4C1	Tribolium castaneum	66.70%	7.70%	9.80%	XP_966563.2
		cytochrome P450 monooxigenase CYP4Q3	Tribolium castaneum	70.30%	7.40%	9.30%	NP_001107847.1
**Probable multidrug resistance-associated lethal(2)03659**	**DN63738_c2_g1**	probable multidrug resistance-associated protein lethal(2)03659	Anoplophora glabripennis	67.20%	24.10%	98.30%	XP_018565536.1
		probable multidrug resistance-associated protein lethal(2)03659	Anoplophora glabripennis	67.60%	23.50%	99.60%	XP_018565531.1
		probable multidrug resistance-associated protein lethal(2)03659 isoform X3	Anoplophora glabripennis	67.00%	29.20%	100.50%	XP_018565520.1
		probable multidrug resistance-associated protein lethal(2)03659 isoform X2	Anoplophora glabripennis	67.00%	26.20%	100.50%	XP_018565518.1
		probable multidrug resistance-associated protein lethal(2)03659 isoform X2	Anoplophora glabripennis	67.00%	26.20%	100.50%	XP_018565519.1
**Probable multidrug resistance-associated lethal(2)03659 isoform X1**	**DN48864_c1_g1**	probable multidrug resistance-associated protein lethal(2)03659	Anoplophora glabripennis	80.10%	80.00%	69.60%	XP_018572717.1
		multidrug resistance-associated protein 4-like	Dendroctonus ponderosae	76.10%	80.00%	71.20%	XP_019763391.1
		probable multidrug resistance-associated protein lethal(2)03659 isoform X1	Tribolium castaneum	73.00%	79.40%	69.10%	XP_015835858.1
		probable multidrug resistance-associated protein lethal(2)03659 isoform X3	Tribolium castaneum	73.00%	95.70%	69.10%	XP_008194033.2
		probable multidrug resistance-associated protein lethal(2)03659 isoform X1	Agrilus planipennis	70.40%	78.50%	68.80%	XP_018326342.1
**Pupal cuticle 20-like**	**DN44960_c0_g1**	pupal cuticle protein 20-like	Anoplophora glabripennis	91.50%	46.70%	30.10%	XP_018564030.1
		pupal cuticle protein 20-like	Dendroctonus ponderosae	92.80%	36.90%	31.60%	XP_019761929.1
		pupal cuticle protein 36-like, partial	Aethina tumida	88.80%	36.80%	30.40%	XP_019877901.1

Blast2Go transcript designation	Trinity transcript ID	Sequences Producing Significant Alignments	Scientific Taxonomy	Sim	Hsp/ HIT	Hsp/ QUERY	NCBI Accession
**Pupal cuticle 20-like**	**DN44960_c0_g1**	pupal cuticle protein 20-like	Dendroctonus ponderosae	80.00%	67.10%	31.30%	XP_019761928.1
		cuticle protein 3-like isoform X2	Dendroctonus ponderosae	83.00%	46.50%	30.10%	XP_019761850.1
**Pupal cuticle 20-like**	**DN59030_c2_g1**	pupal cuticle protein 20-like	Aethina tumida	48.60%	63.00%	43.60%	XP_019867111.1
		pupal cuticle protein 20-like	Aethina tumida	48.60%	63.00%	43.60%	XP_019880001.1
		larval cuticle protein LCP-30-like	Nicrophorus vespilloides	37.00%	94.40%	64.50%	XP_017785749.1
		larval cuticle protein LCP-30-like	Anoplophora glabripennis	40.90%	79.10%	64.80%	XP_018574347.1
		hornerin-like	Nicrophorus vespilloides	40.10%	43.50%	63.90%	XP_017785751.1
**UDP-glucuronosyltransferase 2B7-like**	**DN61595_c0_g3**	UDP-glucuronosyltransferase 2B7-like	Anoplophora glabripennis	82.80%	90.30%	62.10%	XP_018572801.1
		UDP-glucuronosyltransferase 2B30-like isoform X1	Dendroctonus ponderosae	73.80%	98.10%	67.70%	XP_019771209.1
		2-hydroxyacylsphingosine 1-beta-galactosyltransferase-like	Aethina tumida	75.20%	90.20%	63.50%	XP_019876863.1
		UDP-glucuronosyltransferase 2B7-like	Aethina tumida	71.20%	99.40%	62.90%	XP_019866233.1
		UDP-glucuronosyltransferase 2B9	Tribolium castaneum	70.40%	96.50%	65.30%	XP_969004.1
**Uncharacterized oxidoreductase**	**DN44684_c0_g1**	uncharacterized protein LOC108913205	Anoplophora glabripennis	71.30%	97.00%	62.60%	XP_018574226.1
		LOW QUALITY PROTEIN: uncharacterized protein LOC109607529	Aethina tumida	70.40%	90.70%	64.20%	XP_019879569.1
		uncharacterized protein LOC108908201	Anoplophora glabripennis	70.80%	86.40%	63.30%	XP_018567659.1
		uncharacterized protein LOC108908201	Anoplophora glabripennis	70.80%	86.40%	63.30%	XP_018567668.1
		uncharacterized oxidoreductase YjmC	Tribolium castaneum	69.60%	90.80%	63.20%	XP_008195564.1
**Venom carboxylesterase-6-like**	**DN48501_c1_g1**	esterase FE4-like, partial	Anoplophora glabripennis	79.80%	42.90%	55.30%	XP_018572488.1
		venom carboxylesterase-6-like	Anoplophora glabripennis	76.20%	37.00%	63.50%	XP_018572491.1
		esterase E4-like	Anoplophora glabripennis	74.40%	36.40%	63.80%	XP_018572490.1
		venom carboxylesterase-6-like	Anoplophora glabripennis	75.20%	40.60%	63.50%	XP_018572487.1
		esterase E4	Dendroctonus ponderosae	76.80%	32.60%	55.90%	XP_019761960.1
