# Supplementary material for: Transcriptomic analysis reveals similarities in genetic activation of detoxification mechanisms resulting from imidacloprid and chlorothalonil exposure
Source: PLoS One. 2018 Oct 25;13(10):e0205881. doi: 10.1371/journal.pone.0205881 (PMC6201883; doi:10.1371/journal.pone.0205881)
Supplement: S2 Table — Primer efficiencies and BLAST match from NCBI. (PDF) [file pone.0205881.s003.pdf]

S2 Table. Forward and reverse primers used in quantitative PCR. Primer efficiencies and BLAST match from NCBI.

	Forward Primer (5’-3’)	Reverse Primer (5’-3’)	Primer Efficiency	Transcript BLAST x Result	NCBI Accession Numbers
RP4 Reference	AAAGAAACGAGCATTGCCCTTCCG	TTGTCGCTGACACTGTAGGGTTGA	1.93	NA	NA
DN61141	TGCTGAAAGACCTGGAAGTG	CTCATCATGGGAAGAAGACTGG	2.01	cytochrome P450 6k1 isoform X1	XP_970485.1
DN61595	TCCGCAACCTCCATCTTTATAC	AACCTGACCCAAAGATAGTACAG	1.88	UDP-glucuronosyltransferase 2B7-like	XP_018572801.1
DN52191	TCTCCGAAACAGCACTTACAG	ACCTTCTGAGATGCTTGATACTG	1.98	acetylcholine receptor subunit alpha-like	XP_018577209.1
DN45930	CTCACACTGTGGCTTTCTTATTG	TCACACAGGCTTGCAGATAC	2.09	probable cytochrome P450	XP_019770320.1
