# Supplementary material for: Transcriptomic analysis reveals similarities in genetic activation of detoxification mechanisms resulting from imidacloprid and chlorothalonil exposure
Source: PLoS One. 2018 Oct 25;13(10):e0205881. doi: 10.1371/journal.pone.0205881 (PMC6201883; doi:10.1371/journal.pone.0205881)
Supplement: S3 Table — (PDF) [file pone.0205881.s004.pdf]

S3 Table. Gene ontology terms determined from over-expressed transcripts in the imidacloprid, chlorothalonil, and effect group.

GO ID	GO Name	GO Category	P value
Imidacloprid			
GO:0005506	iron ion binding	MOLECULAR FUNCTION	0.020
GO:0006508	proteolysis	BIOLOGICAL PROCESS	0.041
GO:0070011	peptidase activity, acting on L-amino acid peptides	MOLECULAR FUNCTION	0.036
GO:0046906	tetrapyrrole binding	MOLECULAR FUNCTION	0.020
GO:0046914	transition metal ion binding	MOLECULAR FUNCTION	0.041
GO:0004252	serine-type endopeptidase activity	MOLECULAR FUNCTION	0.031
GO:0004175	endopeptidase activity	MOLECULAR FUNCTION	0.031
GO:0008236	serine-type peptidase activity	MOLECULAR FUNCTION	0.031
GO:0008233	peptidase activity	MOLECULAR FUNCTION	0.041
GO:0017171	serine hydrolase activity	MOLECULAR FUNCTION	0.031
GO:0008152	metabolic process	BIOLOGICAL PROCESS	0.016
GO:0003824	catalytic activity	MOLECULAR FUNCTION	0.005
GO:0020037	heme binding	MOLECULAR FUNCTION	0.020
GO:0016705	oxidoreductase activity, acting on paired donors, with incorporation or reduction of molecular oxygen	MOLECULAR FUNCTION	0.015
Chlorothalonil			
GO:0016740	transferase activity	MOLECULAR FUNCTION	0.012445
GO:0016741	transferase activity, transferring one-carbon groups	MOLECULAR FUNCTION	0.009968
GO:0008168	methyltransferase activity	MOLECULAR FUNCTION	0.009968
GO:0003824	catalytic activity	MOLECULAR FUNCTION	0.042288
GO:0032259	methylation	BIOLOGICAL PROCESS	0.009968
Effect Vs. No Effect			
GO:0003824	catalytic activity	MOLECULAR FUNCTION	0.001199
GO:0008152	metabolic process	BIOLOGICAL PROCESS	0.00479

GO:0005506	iron ion binding	MOLECULAR FUNCTION	0.026362
GO:0016772	transferase activity, transferring phosphorus-containing groups	MOLECULAR FUNCTION	0.013269
GO:0016740	transferase activity	MOLECULAR FUNCTION	0.00777
GO:0070011	peptidase activity, acting on L-amino acid peptides	MOLECULAR FUNCTION	0.045676
GO:0046906	tetrapyrrole binding	MOLECULAR FUNCTION	0.026362
GO:0004252	serine-type endopeptidase activity	MOLECULAR FUNCTION	0.039281
GO:0004175	endopeptidase activity	MOLECULAR FUNCTION	0.039281
GO:0008236	serine-type peptidase activity	MOLECULAR FUNCTION	0.039281
GO:0017171	serine hydrolase activity	MOLECULAR FUNCTION	0.039281
GO:0020037	heme binding	MOLECULAR FUNCTION	0.026362
GO:0016705	oxidoreductase activity, acting on paired donors, with incorporation or reduction of molecular oxygen	MOLECULAR FUNCTION	0.019837
