# Supplementary material for: Transcriptomic analysis reveals similarities in genetic activation of detoxification mechanisms resulting from imidacloprid and chlorothalonil exposure
Source: PLoS One. 2018 Oct 25;13(10):e0205881. doi: 10.1371/journal.pone.0205881 (PMC6201883; doi:10.1371/journal.pone.0205881)
Supplement: S4 Table — (PDF) [file pone.0205881.s005.pdf]

S4 Table. Mean CT values determined by quantitative PCR used to calculate transcript expression.

	**Control (leaf disk only)**	**Fungicide (chlorothalonil)**	**Insecticide (imidacloprid)**	**Effect Group**
	**Mean CT ±SD**	**Mean CT± SD**	**Mean CT± SD**	**Mean CT±SD**
RP4 (Reference for DN61141*) Transcriptome*	18.78±0.21	18.69±0.33	19.11±0.79	NA
DN61141 (cytochrome P450 6k1 isoform X1) *Transcriptome*	32.64±3.79	27.08±1.95	29.81±5.33	NA
RP4 (Reference for DN61595*) Transcriptome*	18.83±0.23	18.73±0.36	19.45±0.88	NA
DN61595 (UDP-glucuronosyltransferase 2B7-like) *Transcriptome*	28.92±1.67	25.95±2.66	26.32±3.00	NA
RP4 (Reference for DN52191*) Transcriptome*	18.53±0.27	NA	NA	19.35±0.53
DN52191 (acetylcholine receptor subunit alpha-like) *Transcriptome*	33.99±2.35	NA	NA	22.15±0.77
RP4 (Reference for DN45930*) Transcriptome*	18.38±0.24	NA	NA	19.23±0.53
DN45930 (probable cytochrome P450) *Transcriptome*	30.15±0.73	NA	NA	21.40±0.69
RP4 (Reference for DN61141*) Treatment 2*	21.73±1.97	20.59±1.06	20.33±0.43	NA
DN61141 (cytochrome P450 6k1 isoform X1) *Treatment 2*	30.67±0.50	24.66±1.11	29.04±4.08	NA
RP4 (Reference for DN61595*) Treatment 2*	22.61±1.89	21.97±1.68	21.22±0.46	NA
DN61595 (UDP-glucuronosyltransferase 2B7-like) *Treatment 2*	31.24±1.95	27.16±2.19	29.49±3.81	NA
RP4 (Reference for DN61141*) Treatment 3*	20.95±0.71	21.33±0.83	24.03±2.05	NA
DN61141 (cytochrome P450 6k1 isoform X1) *Treatment 3*	32.61±3.03	29.43±4.62	37.33±1.14	NA
RP4 (Reference for DN61595*) Treatment 3*	21.23±0.81	21.59±0.86	24.03±2.05	NA
DN61595 (UDP-glucuronosyltransferase 2B7-like) *Treatment 3*	32.19±1.26	26.14±2.61	33.01±3.41	NA
